# Phlegmon of the Hand

**Published:** 1897-04

**Authors:** 


### Phlegmon of the Hand.

Phlegmon of the hand is frequently followed by considerable disability, which may become permanent. This is due to the binding together of the soft parts by contracting scar-tissue, and the process may go so far as to cause ankylosis, and even subluxation of joints. It is possible, in a great measure, to avoid this unwished-for result by insisting upon active and passive motions from the time of the very beginning of the healing process. The frequency of the motions is more important than the force exerted. Pain after the exercises shows that they have been too vigorous. The joints should be moved several minutes at a time, amounting in all to two or three hours in the course of a day. The patient himself can usually carry out this treatment if the dressings are properly arranged. Do not wait until the wounds are healed, or your patient may be irreparably disabled.—*International Journal of Surgery.*

